# Fluorescence molecular imaging technology: a promising new strategy for the diagnosis and treatment of gynecologic tumors

**DOI:** 10.3389/fbioe.2025.1774573

**Published:** 2026-01-21

**Authors:** Kailang Li, Bifan Huang, Jin Jiang

**Affiliations:** 1 Department of Radiology, Third People’s Hospital of Xindu District, Chengdu, China; 2 Department of Radiology, Sichuan Provincial People’s Hospital, University of Electronic Science and Technology of China, Chengdu, China

**Keywords:** fluorescence molecular imaging, gynecologic tumors, intraoperative navigation, molecular probes, precision medicine

## Abstract

Gynecologic malignancies such as ovarian, endometrial, and cervical cancers are characterized by challenges in early diagnosis and high therapeutic complexity, creating an urgent need for more precise imaging techniques. Fluorescence molecular imaging, a modality with high sensitivity and high spatial resolution, has demonstrated considerable value in tumor diagnosis and therapy in recent years. Its application in gynecologic oncology is rapidly expanding. By using tumor-specific molecular probes to label neoplastic tissues, this technology enables real-time intraoperative navigation and visualization of tumor lesions and their local features, thereby significantly improving surgical accuracy and therapeutic outcomes. This review summarizes the basic principles of fluorescence imaging, recent advances in molecular probe design, and developments in imaging devices. It places particular emphasis on the value of fluorescence molecular imaging in the diagnosis and precision treatment of gynecologic tumors, aiming to provide systematic theoretical guidance and technical support for related research and clinical practice.

## 1 Introduction

Gynecologic malignancies, including ovarian, endometrial, and cervical cancers—are common yet complex types of cancer in women. Their highly heterogeneous biological behaviors and clinical manifestations pose significant challenges for early diagnosis and effective treatment. Although conventional imaging modalities such as computerized tomography (CT) and magnetic resonance imaging (MRI) play essential roles in tumor localization and morphological assessment, their resolution and specificity often fall short in detecting small or early-stage lesions. In particular, the identification of intraoperative tumor margins and micro-metastatic foci remains inadequately addressed and requires more advanced imaging techniques to supplement existing methods (cf. Masselli and Bourgioti, 2025; Kitajima, 2025). Fluorescence molecular imaging (FMI), an emerging molecular imaging modality, has gained increasing attention in the diagnosis and treatment of tumors in recent years. It offers marked advantages in achieving highly sensitive and highly specific detection of tumor tissues, thus providing new strategies to address current clinical challenges. By designing fluorescent probes targeting tumor-specific biomarkers, FMI enables precise identification and real-time dynamic visualization of tumor cells and their microenvironment, thereby assisting clinicians in accurate diagnosis and the formulation of individualized treatment strategies (cf. Kashihara et al., 2021; Pal et al., 2022). These developments are driving tumor diagnosis and therapy toward greater precision (cf. Grose et al., 2020).

With rapid advances in molecular probe engineering and imaging hardware, FMI has shown substantial advantages in real-time surgical guidance and treatment response assessment. The development of near-infrared (NIR) and second near-infrared window (NIR-II) probes has enhanced tissue penetration depth and imaging signal-to-noise ratio, making high-resolution visualization of the tumor microenvironment and vascular networks possible (cf. He et al., 2022; Hu et al., 2020). In addition, “activatable’’ fluorescent probes based on tumor-specific enzymatic activity can suppress background signals and achieve high-contrast tumor imaging, further improving diagnostic sensitivity and specificity (cf. He et al., 2025). These innovations not only facilitate more precise tumor resection—reducing intraoperative misresection and residual disease—but also enable dynamic monitoring of therapeutic outcomes, providing strong support for postoperative evaluation (cf. Hou et al., 2026; Rainu et al., 2023).

Importantly, FMI combines highly selective molecular probes with advanced imaging devices to clearly distinguish tumor tissue from normal structures during surgery, effectively addressing the stringent requirements for real-time and highly accurate imaging in gynecologic oncology. Fluorescent probes targeting tumor-related molecules such as epidermal growth factor receptor and vascular endothelial growth factor have demonstrated excellent imaging performance in various clinical studies and hold promise for early detection and precise surgical navigation in gynecologic cancers (cf. Pal et al., 2022). Furthermore, the development of multimodal molecular probes—those integrating both fluorescence and MRI functions-has the potential to further enhance tumor localization accuracy and multidimensional analysis capabilities (cf. Zhao et al., 2020; Zou et al., 2024).

**FIGURE 1:**
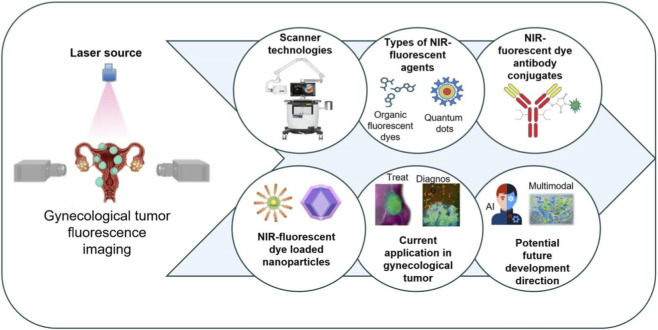
An illustration that represents fluorescence molecular imaging and their key applications in the diagnosis and treatment of gynecologic tumors, including types of NIR-fluorescent agents, NIR-fluorescent dye antibody conjugates, and current application in gynecological tumor et al. This figure was created with Biorender.com.

This review synthesizes recent applications of FMI in gynecologic cancers, summarizing its basic principles, advances in molecular probe design, and progress in imaging systems (Figure 1), with an emphasis on its utility in intraoperative navigation and enhanced therapeutic targeting, to provide systematic theoretical guidance and technical support for research and clinical application.

## 2 Principles, components, and current development of fluorescence molecular imaging technology

### 2.1 Basic principles of fluorescence imaging

Fluorescence imaging is based on the intrinsic properties of fluorescent molecules. When these molecules absorb excitation light of a specific wavelength, they transition to an excited state; upon returning to their ground state, they emit fluorescence with a longer wavelength. This process enables specific labeling and detection of target molecules and forms the core mechanism of fluorescence imaging. The selective binding capability of fluorescent probes, combined with distinct excitation–emission spectra, allows fluorescence imaging to achieve high sensitivity and high contrast within biological tissues, thereby visualizing cellular or tissue structures and functional states with clarity.

Excitation light sources typically include lasers or high-intensity light-emitting diode (LED) systems, with wavelengths precisely tuned to the excitation peak of the selected fluorophore to ensure efficient excitation. In imaging systems, detectors-such as photomultiplier sensors-capture fluorescence signals with high sensitivity. Imaging platforms such as confocal microscopes, two-photon microscopes, and fluorescence lifetime imaging microscopy employ different optical configurations and signal-processing strategies to enhance spatial resolution, signal-to-noise ratio, and imaging depth. For example, two-photon fluorescence imaging utilizes nonlinear two-photon absorption processes in which longer-wavelength photons are used for excitation. This results in deeper tissue penetration, reduced photodamage, and intrinsic optical sectioning capability, enabling high-contrast imaging of deeper layers of living tissues (cf. Feng et al., 2021).

In addition, modern fluorescence imaging technologies have incorporated excitation–emission matrix imaging, which enables simultaneous detection and differentiation of multiple fluorescent molecules within complex biological samples by scanning across multiple excitation and emission wavelengths (cf. Katz et al., 2023). Fluorescence lifetime imaging, which measures the emission lifetime of fluorescent molecules, provides richer dynamic information regarding molecular environments and interactions (cf. Peng, 2021; Ullah et al., 2024). Overall, the imaging system functions through a coordinated mechanism: the excitation source emits light at a specific wavelength to excite fluorescent molecules, which then emit fluorescence. The detector collects these signals, which are subsequently processed through optical filtering and electronic signal algorithms to generate high-resolution fluorescence images that reflect the spatial distribution and functional status of molecules and cells.

### 2.2 Design and classification of fluorescent probes

Fluorescent probes, as a central component of fluorescence molecular imaging, directly determine the sensitivity, specificity, and scope of imaging applications. In biomedical fields such as gynecologic oncology, the rational design of fluorescent probes not only enhances early tumor detection and intraoperative navigation accuracy but also drives advances in molecular-targeted therapies. Fluorescent probes are generally classified into two major categories: traditional small-molecule fluorescent dyes and emerging nanoprobes, each possessing distinct advantages and limitations. Traditional small-molecule fluorescent dyes-such as rhodamines, acridinium esters, BODIPY derivatives, and quinolone-based dyes-exhibit well-defined structures, mature synthesis pathways, and excellent optical properties (e.g., high quantum yield, large Stokes shift, and strong photostability). Their chemical structures can be readily modified to achieve functionalization for diverse biological imaging needs. By introducing specific functional groups or structural units, their spectral characteristics and biocompatibility can be fine-tuned, enabling selective imaging of intracellular organelles (cf. Jun et al., 2020; Jiang et al., 2024). For instance, BODIPY-based probes, known for their exceptional photostability and tunable photophysical features, are widely employed for imaging organelles and biomolecules (cf. Zhang et al., 2024). Nanoprobes-such as carbon quantum dots, sulfur quantum dots, metallic nanoparticles, and self-assembled nanoprobes-have emerged as a major research focus in recent years. These nanoprobes offer advantages including small size, large surface area, ease of surface functionalization, and outstanding optical properties such as broad excitation spectra, narrow emission bands, and high photostability. They also allow multimodal imaging and exhibit favorable biocompatibility compatible with complex biological environments (cf. Kadian et al., 2023). Among them, self-assembled nanoprobes, designed through molecular self-assembly strategies, can respond to aberrant enzyme activities, acidic pH, or reactive species within the tumor microenvironment, thereby enabling high specificity and signal amplification for cancer diagnostics (cf. Wu W. et al., 2025).

Targeting tumor-associated molecules is a key design strategy for improving probe selectivity. Common approaches include antibody conjugation, peptide-based targeting, and small-molecule ligand binding. Antibody-conjugated fluorescent probes utilize the high affinity of antibodies for tumor antigens, but are limited by their large molecular weight, poor tissue penetration, and potential immunogenicity (cf. Chen et al., 2022). Peptide-based targeting probes-such as RGD peptides targeting integrin receptors-offer advantages of small size, high affinity, ease of modification, and favorable biocompatibility (cf. Schreiber et al., 2022). Additionally, small-molecule targeting probes are designed to recognize tumor-specific metabolites or receptors and incorporate fluorescent dyes to achieve *in vivo* tumor-specific imaging. Examples include probes targeting carbonic anhydrase IX or breast cancer-related proteins, which feature rapid clearance and minimal immunogenicity (cf. Zhang et al., 2021).

### 2.3 Development trends in imaging devices

The development of imaging equipment has progressed from traditional *ex vivo* fluorescence microscopes to *in vivo* real-time imaging systems, while the emergence of portable and surgery-dedicated devices has further accelerated clinical translation and expanded applications. Traditional fluorescence microscopes, primarily used for observing cells and tissue sections in vitro, offer high spatial resolution and strong optical imaging performance. However, their limited imaging depth and inability to provide real-time dynamic observations restrict their utility in tracking and localizing tumors within the complex *in vivo* environment. To address these limitations, researchers have developed techniques such as NIR optical microscopy and multiphoton excitation fluorescence imaging. These modalities leverage the deeper tissue penetration, reduced scattering, and lower absorption of NIR light to visualize deeper anatomical structures while maintaining high spatial resolution and tissue contrast. For example, two-photon excitation microscopy combined with NIR-emissive probes enables high-resolution imaging in deep tissues, and the introduction of thermally activated delayed fluorescence probes further improves signal-to-noise ratio and imaging accuracy (cf. Wang et al., 2024). In addition, macroscopic fluorescence lifetime imaging systems that integrate modulation transfer functions and structured illumination can effectively separate superficial and deep fluorescence signals, enhancing depth selectivity and the biological interpretability of imaging data (cf. Yuan et al., 2025). These advancements collectively signify the shift of fluorescence imaging devices from simple *ex vivo* observation toward sophisticated *in vivo* real-time imaging.

As surgical procedures increasingly demand real-time navigation, the development of portable and surgery-dedicated fluorescence imaging instruments has become a major research focus. Portable devices, known for their compact size, ease of use, and low cost, are suitable for rapid intraoperative assessment and margin detection. For instance, smartphone-based fluorescence imaging and spectroscopic systems have been applied successfully in the real-time detection of tumor margins during breast cancer surgery, demonstrating high sensitivity and classification accuracy (cf. Thapa et al., 2023). Furthermore, NIR fluorescence imaging systems combined with targeted nanoprobes offer high-contrast tumor visualization during gynecologic oncology surgery, effectively improving complete resection rates and clinical outcomes (cf. Polom et al., 2021; Solidoro et al., 2024).

Future trends in imaging device technology will likely center on multimodal integration and intelligent systems. Multimodal imaging, which merges fluorescence imaging with techniques such as photoacoustic imaging or magnetic resonance imaging, provides comprehensive structural and functional information that greatly enhances diagnostic accuracy and surgical precision (cf. Daneshpour et al., 2025). Artificial intelligence (AI) is expected to play a transformative role in image processing, feature extraction, and surgical decision-making, promoting the development of automated and intelligent imaging systems (cf. Wendler et al., 2025). Moreover, the integration of microfluidics and 3D-printing technologies offers new possibilities for fabricating miniaturized and customized imaging platforms, further advancing portability and device personalization (cf. Elkawad et al., 2022).

## 3 Applications of fluorescence imaging in the diagnosis of gynecologic malignancies

### 3.1 Value in early diagnosis

Fluorescence imaging technology demonstrates substantial value in the early diagnosis of tumors, particularly in the highly sensitive detection of small lesions. For example, studies have shown that γ-glutamyl transpeptidase is overexpressed in ovarian cancer, and a responsive fluorescent probe, Py-GSH, can be used for rapid tumor detection. This probe demonstrates excellent selectivity and rapid responsiveness both *in vivo* and *in vitro* (cf. Zhou et al., 2019). In the diagnosis of uterine tumors, photoacoustic imaging (PAI) has also shown considerable potential. Using non-ionizing radiation to detect pathological changes in uterine tissues, PAI can effectively distinguish healthy tissues from benign tumors (e.g., fibroids) and malignant tumors (e.g., endometrial carcinoma). By detecting differences in acoustic intensity, PAI provides direct information related to tumor properties, improving diagnostic accuracy (cf. Walhikmah et al., 2024). Moreover, NIR fluorescence imaging has been widely applied for intraoperative navigation in gynecologic oncology. It enables real-time visualization of tumor tissues and critical anatomical structures during surgery, facilitating more precise tumor resection and lymph node identification (cf. Handgraaf et al., 2014). For instance, in ovarian cancer surgery, folate receptor-α–targeted fluorescence imaging has been shown to enhance surgical staging and cytoreductive procedures, thereby improving patient outcomes (cf. van Dam et al., 2011).

From a diagnostic strategy perspective, combining fluorescence imaging with serum tumor biomarkers to create a multimodal diagnostic system significantly enhances sensitivity and specificity for early detection. Although serum biomarkers such as CA125 are commonly used in gynecologic cancer screening, their diagnostic performance is often limited by high false-positive and false-negative rates. Integrating biomarker measurements with fluorescence imaging allows the incorporation of both molecular-level and tissue-level information. For example, fluorescent probes targeting specific proteases or enzymes, combined with biomarker detection, can enable earlier identification of tumor development-even before abnormalities appear on conventional imaging. This multimodal diagnostic approach provides a new pathway for early cancer detection and supports the development of precision and personalized medicine (cf. Wu Z. et al., 2025; Cuadrado et al., 2024).

### 3.2 Imaging analysis of tumor heterogeneity and molecular characteristics

Tumor heterogeneity refers to the diversity of tumor cells at the molecular, morphological, and functional levels. This heterogeneity exists both between tumors from different individuals and within a single tumor, and it significantly affects tumor diagnosis, treatment response, and prognosis. Due to its high sensitivity and molecular specificity, fluorescence molecular imaging has become an important tool for elucidating tumor heterogeneity, particularly in gynecologic oncology research and clinical practice. In terms of revealing molecular heterogeneity among tumor cell subpopulations with multiplex fluorescent probes, studies have demonstrated that multicolor fluorescence probes can simultaneously label multiple molecular targets within the same tumor tissue, allowing differentiation and spatial mapping of various tumor cell subtypes. For example, combining fluorescent probes targeting epidermal growth factor recepto, estrogen receptor, and human epidermal growth factor receptor 2 allows multiplex labeling within the same tissue section, revealing the molecular complexity and spatial heterogeneity of the tumor (cf. Chen et al., 2023; Xie et al., 2025). Furthermore, fluorescence-lifetime imaging microscopy (FLIM), which measures the fluorescence lifetime of endogenous metabolic cofactors such as NAD(P)H, enables label-free detection of metabolic heterogeneity, offering a novel approach for characterizing metabolic diversity among tumor cell populations (cf. Shirshin et al., 2022). Together, these techniques enable precise identification of molecular and metabolic features of different intratumoral subpopulations, forming a key foundation for precision oncology.

**FIGURE 2:**
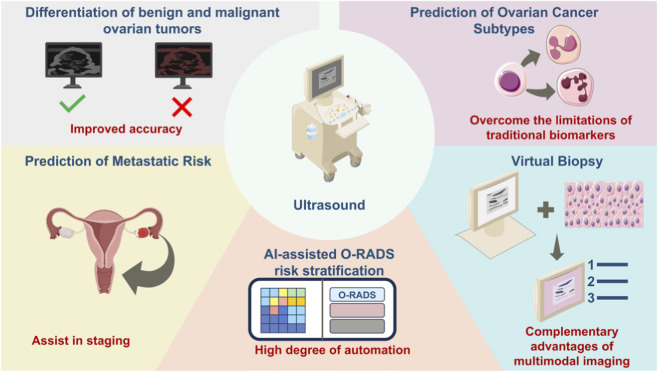
Applications of ultrasound-based radiomics in ovarian cancer management. The major clinical applications of ultrasound-based radiomics in ovarian cancer, including tumor differentiation, AI-assisted O-RADS risk stratification, subtype prediction, metastatic risk assessment, and virtual biopsy. By integrating imaging features with artificial intelligence and clinical data, these approaches enhance diagnostic accuracy, automate risk classification, and support individualized treatment planning. Reproduced with permission from (cf. Huang et al., 2025), copyright 2025 ©The Author(s).

Regarding imaging differences among gynecologic tumor subtypes and their clinical implications, fluorescence molecular imaging also demonstrates significant advantages. Ovarian cancer and endometrial cancer are characterized by substantial molecular heterogeneity, with different subtypes exhibiting distinct biomarker expression profiles, therapeutic responses, and prognoses. Radiogenomics, which integrates CT, MRI, or positron emission tomography imaging with gene expression data, has successfully identified imaging phenotypes associated with BRCA mutations, homologous recombination deficiency, and immune-related biomarkers in ovarian cancer, as well as POLE mutations, microsatellite instability and tumor mutational burden in endometrial cancer, such as ultrasound radiomics is used in tumor differentiation, O-RADS risk stratification, subtype prediction, metastasis risk assessment and virtual biopsy of ovarian cancer (Figure 2) (cf. Huang et al., 2025). In the domain of molecular imaging probes, fluorescently labeled antibodies and nanoprobes targeting EGFR, folate receptor-α (FRα), EpCAM, and other biomarkers enable precise localization and molecular imaging of different tumor subtypes, supporting intraoperative navigation.

## 4 The auxiliary role of fluorescence molecular imaging in the treatment of gynecologic tumors

### 4.1 Precision guidance for treatment

Utilizing fluorescence imaging technology to achieve precise differentiation between tumor and normal tissues-thereby assisting surgeons in accomplishing complete tumor resection-represents a highly promising direction in contemporary oncologic surgery. Traditional operations rely on intraoperative frozen-section pathology; however, the positive rate of surgical margins is at a relatively high level, leading to increased local recurrence and poorer prognoses. This issue is particularly prominent in gynecologic oncology, where residual tumor tissue markedly elevates the risk of recurrence and adversely affects long-term survival and quality of life. Conventional surgical assessments depend on visual inspection and palpation to determine tumor boundaries, but these approaches often fail to clearly distinguish tumor from normal tissue, frequently resulting in positive margins and residual disease. The emergence of fluorescence molecular imaging offers a powerful solution to this limitation. By specifically labeling tumor cells, fluorescence imaging enables real-time and dynamic intraoperative visualization of tumor margins, substantially enhancing the precision of tumor resection.

Near-infrared fluorescence imaging using indocyanine green (ICG) has demonstrated notable advantages in gynecologic oncology. ICG is primarily employed for sentinel lymph node mapping in gynecologic surgeries-particularly for endometrial and cervical cancers-where it is regarded as a feasible, safe, time-efficient, and reliable lymphatic tracer. In addition, ICG fluorescence imaging facilitates ureteral identification, thereby reducing the risk of iatrogenic ureteral injury during gynecologic procedures (cf. Incognito et al., 2022; Huang et al., 2022). In the targeted therapy of ovarian cancer, fluorescence imaging also shows significant potential. Studies have demonstrated that intraoperative tumor-specific fluorescence imaging using folate receptor-α–targeted fluorescent agents can improve surgical staging and enhance cytoreductive outcomes, ultimately leading to better prognoses (cf. Nougaret et al., 2021; Zapardiel et al., 2021). Another study further reported that fluorescence imaging exhibits high sensitivity in detecting microlesions in ovarian cancer, especially when 5-aminolevulinic acid (5-ALA) is used, thereby markedly improving diagnostic accuracy (cf. Erdemoglu et al., 2025). Meanwhile HER2-targeted magnetic iron oxide nanoparticles conjugated with cisplatin have shown powerful antitumor effects in ovarian cancer xenograft models (cf. Satpathy et al., 2019). In summary, FMI holds substantial potential for improving surgical completeness and facilitating targeted treatment strategies in gynecologic oncology.

### 4.2 Postoperative therapeutic evaluation and recurrence monitoring

Postoperative therapeutic evaluation and recurrence monitoring are essential components of gynecologic cancer management. Timely and accurate detection of residual disease and early recurrence is crucial for optimizing treatment strategies and improving patient outcomes. FMI, owing to its high sensitivity and real-time imaging capabilities, serves as a powerful tool for postoperative monitoring. Using specific fluorescent probes to label tumor-associated molecules enables rapid identification of small lesions after surgery, assisting clinicians in assessing surgical margins and detecting potential residual tumor cells, thereby informing subsequent therapeutic decisions. For patients with advanced gynecologic tumors, even after extensive pelvic surgery aimed at achieving negative margins, vigilant postoperative surveillance is necessary to prevent recurrence. FMI can aid in localizing lesions postoperatively, allowing evaluation of tumor size, extent, and residual disease. Its high sensitivity is particularly valuable during early recurrence, guiding decisions for secondary surgery or adjusting adjuvant therapy protocols (cf. Ubinha et al., 2024).

Moreover, FMI can assist in evaluating therapeutic response, particularly during postoperative chemotherapy or radiotherapy. By dynamically monitoring tumor activity and changes in micro-metastatic foci, clinicians can adjust treatment plans in real time to achieve precision medicine. In ovarian cancer, tumor stage and residual tumor size strongly influence prognosis; thus, continuous monitoring of fluorescence signals from residual lesions can indicate treatment response and guide therapeutic adjustments (cf. Shen et al., 2023). Among elderly gynecologic oncology patients, postoperative recovery and complication profiles significantly affect long-term outcomes. Fluorescence-assisted recurrence monitoring, combined with clinical indicators, supports individualized risk assessment and therapeutic evaluation, enabling early intervention and optimized recovery management (cf. Chen and Chen, 2025).

## 5 Challenges and future directions of fluorescence molecular imaging technology

FMI represents a significant advance in precision medicine for gynecologic oncology. As a highly sensitive and specific imaging modality, it substantially improves diagnostic accuracy and provides unique advantages in key clinical applications, including intraoperative navigation and targeted therapy. This technology complements conventional imaging by enabling visualization of the tumor microenvironment and real-time dynamic monitoring, offering clinicians more intuitive and reliable decision-making tools. However, its clinical translation faces multiple technical limitations and obstacles, primarily related to probe stability, phototoxicity and tissue penetration depth. Future research should focus on optimizing the physicochemical and photophysical properties of fluorescent probes, enhancing biocompatibility and stability, developing low-phototoxicity excitation strategies and validating deep-penetration probes.
